# Musclin prevents depression-like behavior in male mice by activating urocortin 2 signaling in the hypothalamus

**DOI:** 10.3389/fendo.2023.1288282

**Published:** 2023-12-05

**Authors:** Koji Ataka, Akihiro Asakawa, Haruki Iwai, Ikuo Kato

**Affiliations:** ^1^ Laboratory of Medical Biochemistry, Kobe Pharmaceutical University, Kobe, Japan; ^2^ Department of Psychosomatic Internal Medicine, Kagoshima University Graduate School of Medical and Dental Sciences, Kagoshima, Japan; ^3^ Department of Oral Anatomy and Cell Biology, Kagoshima University Graduate School of Medical and Dental Sciences, Kagoshima, Japan

**Keywords:** musclin, depression, urocortin 2, muscle-brain axis, myokines

## Abstract

**Introduction:**

Physical activity is recommended as an alternative treatment for depression. Myokines, which are secreted from skeletal muscles during physical activity, play an important role in the skeletal muscle-brain axis. Musclin, a newly discovered myokine, exerts physical endurance, however, the effects of musclin on emotional behaviors, such as depression, have not been evaluated. This study aimed to access the anti-depressive effect of musclin and clarify the connection between depression-like behavior and hypothalamic neuropeptides in mice.

**Methods:**

We measured the immobility time in the forced swim (FS) test, the time spent in open arm in the elevated-plus maze (EPM) test, the mRNA levels of hypothalamic neuropeptides, and enumerated the c-Fos-positive cells in the paraventricular nucleus (PVN), arcuate nucleus (ARC), and nucleus tractus solitarii (NTS) in mice with the intraperitoneal (i.p.) administration of musclin. Next, we evaluated the effects of a selective corticotropin-releasing factor (CRF) type 1 receptor antagonist, selective CRF type 2 receptor antagonist, melanocortin receptor (MCR) agonist, and selective melanocortin 4 receptor (MC4R) agonist on changes in behaviors induced by musclin. Finally we evaluated the antidepressant effect of musclin using mice exposed to repeated water immersion (WI) stress.

**Results:**

We found that the i.p. and i.c.v. administration of musclin decreased the immobility time and relative time in the open arms (open %) in mice and increased urocortin 2 (Ucn 2) levels but decreased proopiomelanocortin levels in the hypothalamus. The numbers of c-Fos-positive cells were increased in the PVN and NTS but decreased in the ARC of mice with i.p. administration of musclin. The c-Fos-positive cells in the PVN were also found to be Ucn 2-positive. The antidepressant and anxiogenic effects of musclin were blocked by central administration of a CRF type 2 receptor antagonist and a melanocortin 4 receptor agonist, respectively. Peripheral administration of musclin also prevented depression-like behavior and the decrease in levels of hypothalamic Ucn 2 induced by repeated WI stress.

**Discussion:**

These data identify the antidepressant effects of musclin through the activation of central Ucn 2 signaling and suggest that musclin and Ucn 2 can be new therapeutic targets and endogenous peptides mediating the muscle−brain axis.

## 1 Introduction

Mental health disorders contribute greatly to the global burden of disease and, in 2019, they were the 7th leading cause of disability-adjusted life-years (DALYs) (1). The prevalence of mental disorders has been reported to be 11727.3 cases per 100,000 people in men and 12760.0 cases per 1000,000 people in women (1). Mental disorders include various illnesses, such as depressive disorders, anxiety disorders, bipolar disorder, and schizophrenia; depressive disorders comprise the largest proportion (37.3%) of DALYs of all mental disorders (1). The global prevalence of major depressive disorder has increased owing to the coronavirus disease 2019 (COVID-19) pandemic (2). Severe depression leads to suicidal ideation and suicide attempts (3, 4). The management of depressive episodes is categorized into four therapeutic strategies: generic psychosocial intervention, psychological therapy, pharmacotherapy, and electroconvulsive therapy (5). Although psychological and pharmacological therapies work well for depressive disorders (5), 30.9% of adults with major depressive disorder do not respond to medication (6). Moreover, alternative treatments are important in countries that lack sufficient medicines, doctors, and clinical psychologists with the necessary skills (3). Recently, physical activity has attracted considerable attention as a treatment and preventive measure for depression (7). The World Health Organization (WHO) defines physical activity as any bodily movement produced by skeletal muscles that requires energy expenditure (8). Several studies have demonstrated that exercise or a combination treatment with exercise and antidepressant reduces the symptoms of depression (9). Current guidelines of various committees in the world such as those of the UK National Institute for Health and Clinical Excellence (NICE), the Canadian Network for mood and anxiety treatments (CANMAT), and the Physical Activity Guidelines for Americans 2nd edition have recommended physical activity intervention against depression as an optional treatment (10–12). Furthermore, home-based exercise has been reported to improve depressive symptoms in patients with treatment-resistant major depressive disorder, as assessed by the Hamilton Depression Rating Scale (HAMD) and Beck Depression Inventory (BDI-II) (13).

Skeletal muscles convert chemical energy to mechanical energy to generate power, to bring about movement, and to maintain their position (14). In addition to mechanical function and energy homeostasis, skeletal muscles have endocrine functions and secrete various cytokines and peptides, such as IL-6, IL-8, IL-15, brain-derived neurotrophic factor (BDNF), insulin-like growth factor-1 (IGF-1), fibroblast growth factor 21 (FGF21), follistatin-like protein-1 (FSTL-1), and irisin, which are termed myokines. Myokines are secreted during the contraction of skeletal muscles and alter the function of not only the skeletal muscle itself but also other tissues, such as the liver, bone, pancreas, gastrointestinal tract, and brain (15). Myokines contribute to the effects of physical activity intervention. In the muscle-brain crosstalk induced by physical activity, cathepsin B, which is required for improvement in memory, was found to be released during a running wheel exercise in mice and a treadmill exercise in humans (16). BDNF was found to be released by aerobic exercise; it activates neurogenesis in the hippocampus of participants, which improves cognitive function (17). While the effect of muscle-brain crosstalk on memory and cognitive functions has been demonstrated, irisin has been shown to improve depression in rats (18). In addition, the serum irisin level was negatively correlated with the HAMD scale score in patients with acute ischemic stroke (19).

Musclin has been cloned from skeletal muscle, and it is the same peptide as osteocrin, which is cloned from bone (20). Musclin is a myokine released during physical activity that has inhibitory effects on insulin-stimulated glucose uptake and glycogen synthesis in myocytes; it also demonstrated physical endurance effects in mice (21, 22). In humans, the serum level of musclin was found to be lower in patients with heart failure (23) and higher in patients with type 2 diabetes (24) compared to healthy controls; therefore, various studies have assessed the effects of musclin on heart diseases and insulin resistance (25, 26).

The hypothalamus plays a central role in the regulation of feeding, energy consumption, circadian rhythms, and physical and mental responses to various stressors (27). We previously reported an association between hypothalamic neuropeptides and emotional behaviors in various stress conditions in rodents: occlusal disharmony, anorexia, *Helicobacter pylori* infection, and psychological stress (28–31). Furthermore, hypothalamic and skeletal muscle cross-talk has been recently proposed, in which myokines contribute to the prevention of hyperphagia and becoming overweight, promotion of muscle mitochondrial biogenesis, and body temperature regulation (32).

The aims of the present study were to evaluate the effects of musclin on emotional behavior and the possibility of its clinical application for depression in mice and to reveal the central mechanism of musclin by focusing on the activity of neuropeptides in the hypothalamus.

## 2 Materials and methods

### 2.1 Synthesis and purification of musclin

Musclin (SFSGFGSPLDRLSAGSVEHRGKQRKAVDHSKKRFGIPMDRIGRNRLSSSRG; molecular weight: 5627.4) was synthesized via a solid-phase methodology according to the Fmoc strategy using an automated peptide synthesizer (Model Pioneer, Thermo Fisher Scientific, Waltham, MA, USA). The crude peptide was purified by reverse-phase HPLC (Delta 600 HPLC System, Waters, MA, USA) using a Develosil ODS-HG-5 column (2 × 25 cm, Nomura Chemical Co., Ltd., Aichi, Japan). The purity of the peptide was confirmed using analytical HPLC, matrix-assisted laser desorption/ionization time-of-flight mass spectrometry (MALDI-TOF MS), and amino acid analysis.

### 2.2 Animals

Seven-week-old male C57BL/6J mice were purchased from Japan SLC, Inc. (Shizuoka, Japan). The mice were individually maintained in a pathogen-free facility at 24 ± 2°C and 50 ± 10% humidity, under a 12-h/12-h light/dark cycle (lights on 07:00 a.m. to 07:00 p.m.), with ad libitum access to standard chow (3.4 kcal/g, CE-2; CLEA Japan Inc., Tokyo, Japan) and tap water, in the animal facility of Kobe Pharmaceutical University. All animal experiments were approved by the Kobe Pharmaceutical University Committee for Animal Experiments (approval no. 2023-011 and -014).

### 2.3 Cannula implantation for intracerebroventricular administration

The mice were anesthetized by the intraperitoneal (i.p.) administration of a mixture of 0.3 mg/kg medetomidine, 4.0 mg/kg midazolam, and 5.0 mg/kg butorphanol (1). A guide cannula (25-gauge; Eicom, Kyoto, Japan) was implanted into the right lateral ventricle using a Kopf stereotaxic frame (David Kopf Instruments, Tujunga, CA, USA). The stereotaxic coordinates were 0.8 mm posterior to bregma, 1.5 mm left lateral to the midline, and 1.2 mm below the outer skull surface. The guide cannula was secured with dental cement (Super Bond; Sun Medical Co. Ltd., Moriyama, Japan) and anchored to the dorsal surface of the skull using a stainless-steel screw (AN-3; Eicom). A dummy cannula (AD-4; Eicom) was placed in each guide cannula and fixed using a screw cap (AC-4; Eicom) to prevent occlusion. After cannula implantation, the mice were made to recover from anesthesia by i.p. administration of 0.3 mg/kg atipamezole. The mice were lightly anesthetized by isoflurane inhalation and intracerebroventricular (i.c.v.) administration of drugs was performed 7 days after cannula implantation. The dummy cannulae were replaced with microinjection cannulae (AMI-5; Eicom) that were 1 mm longer than the guide cannulae. Each microinjection cannula was connected to a polyethylene tube (PE-50; Clay Adams, Parsippany, NJ, USA) that was connected to a microsyringe. At the end of the experiments, the mice were euthanized by carbon dioxide inhalation and the correct locations of the i.c.v. cannulae were verified with 10 μl of 0.05% cresyl violet dye.

### 2.4 Measurement of depression-like behavior

To assess the antidepressant effects of i.p. and i.c.v. administration of musclin, we conducted a forced swim (FS) test. The FS test is one of the most commonly used assays for evaluating antidepressant effects in mice, in which the immobility time and the swimming distance are measured as indicators of willingness to escape from an inescapable situation (33). The mice were transferred into the cylinder (diameter, 17 cm; height, 25 cm) with tap water at 25°C. The duration of immobility and swimming distance were measured for the last 4 min of the 6-min test session using a video tracking system (ANY-maze, Stoelting Co. IL, USA).

### 2.5 Measurement of anxiety-like behavior

To assess the effects of i.p. or i.c.v. administration of musclin on anxiety, we conducted the elevated plus-maze (EPM) test, which is a popular screening tool for compounds with anxiolytic potential (34). Mice, which were separated from those that underwent the FS test, were placed at the center of the EPM apparatus and allowed to freely explore the maze for 5 min, to avoid the influence of the FS test. We measured the time spent in open arms and total distance, which are used as indexes of anxiety-like behaviors, using a video tracking system (ANY-maze); the relative time spent in the open arms (open %) was calculated as follows: (time spent in the open arm)/(time spend in the open arm + time in the closed arm) × 100.

### 2.6 Tissue sampling

To avoid the influence of behavioral tests, tissues were sampled from mice other than those undergoing behavioral tests. The mice were anesthetized by i.p. administration of a mixture of 0.3 mg/kg medetomidine, 4.0 mg/kg midazolam, and 5.0 mg/kg butorphanol, perfused with 0.1 M phosphate-buffered saline (pH 7.0), and euthanized by perfusion without awakening from anesthesia. Brain tissues were excised and isolated for RT-qPCR analysis. For immunohistochemistry analysis, the mice were perfused with 0.1 M phosphate-buffered saline (pH 7.0) followed by 4% paraformaldehyde and 0.5% glutaraldehyde in 0.1 M phosphate buffer.

### 2.7 RT-qPCR

Total RNA was extracted from the hypothalamus using an RNeasy Plus Mini Kit (74134; QIAGEN, Hilden, Germany). RNA was reverse transcribed to cDNA using the PrimeScript II 1st Strand cDNA Synthesis Kit (6210A; TAKARA). RT-qPCR was performed using the Power SYBR Green Master Mix (Thermo Fisher Scientific, Waltham, MA, USA) according to the manufacturer’s protocol. Relative mRNA levels were quantified using the 2^–ΔΔCT^ method. A significant change in mRNA expression was defined only when the 2^–ΔΔCT^ value increased by > 2-fold or decreased by < 0.5-fold, in addition to the detection of significance through statistical analyses. The primers used for RT-qPCR were purchased from Takara Bio Inc. (Shiga, Japan) and Bio-Rad (CA, USA) and are listed in **Supplementary Table 1**.

### 2.8 Immunohistochemistry

Coronal sections (25 µm) of the hypothalamus and the nucleus tractus solitarii (NTS) area were obtained using a cryostat (CryoStar NX70; Thermo Fisher Scientific). Hypothalamic sections were incubated with rabbit anti-c-Fos antibody (1:100; ABE457; Merck Millipore, Billerica, MA, USA), mouse anti-c-Fos antibody (1:500; sc-166940; Santa Cruz Biotechnology, Dallas, TX, USA), rabbit anti-urocortin (Ucn) 2 antibody (1:5,000; Y363; Yanaihara Institute Inc., Shizuoka, Japan), and guinea pig anti-product gene protein 9.5 (PGP9.5; 1:1,000; GP14104; Neuromics, Edina, MN, USA), which is a pan-neuronal marker (35, 36), at 4 °C overnight. NTS sections were incubated with rabbit anti-c-Fos antibody (1:100; ABE457) at 4 °C overnight. The sections were then incubated with the secondary antibodies Alexa Fluor 488-conjugated donkey anti-rabbit IgG (1:500; ab150065; Abcam, Cambridge, UK), Alexa Fluor 555-conjugated donkey anti-mouse IgG (1:500; ab150110; Abcam), and Alexa Fluor 647-conjugated donkey anti-guinea pig IgG (1:500; 706-605-148; Jackson ImmunoResearch Labs, West Grove, PA, USA) at 25 °C for 3 h. Nuclei were stained with 4,6-diamidino-2-phenylindole dihydrochloride solution (DAPI; D523; Dojindo Molecular Technologies Inc., Kumamoto, Japan). Images were captured using a confocal laser microscope (LSM 900; Carl Zeiss, Jena, Germany). c-Fos-positive cells in the paraventricular nucleus (PVN), arcuate nucleus (ARC) of the hypothalamus, and NTS were enumerated on one side of each of the six tissue sections per mouse at 200× magnification using confocal laser microscopy. The averages were calculated and used in subsequent analyses. The c-Fos-positive cells were counted manually. Ucn 2 and c-Fos-positive cells, DAPI and c-Fos-positive cells, Ucn 2 and DAPI-positive cells, Ucn 2 and PGP9.5-positive cells, and DAPI and PGP9.5-positive cells in the paraventricular nucleus were observed at 400 × magnification using confocal laser microscopy.

### 2.9 Drug administration

**Figure 1:**
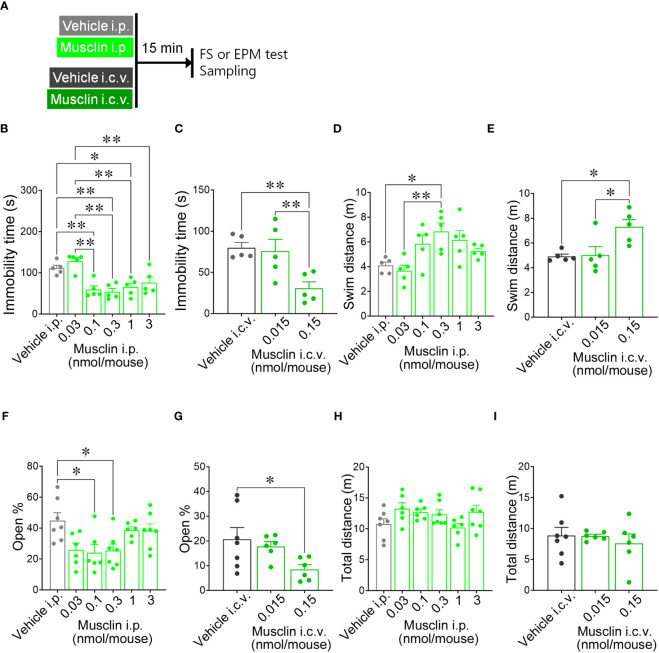
Effects of musclin on the immobility time and the swim distance in the forced swim (FS) test and the open % and the total distance in the elevated-plus maze (EPM) test. **(A)** Mice were administered musclin, and the FS test, EPM test, and tissue sampling were performed according to the schedule. The immobility time **(B, C)** and the swim distance **(D, E)** of mice with administration of musclin were measured in the FS test (*n* = 5). The open % was calculated from time spent in the open arm **(F, G)**, and the total distance **(H, I)** was measured in the EPM test (*n* = 6–7). Data are represented as means ± SEM. Differences were considered significant at *p < 0.05 and **p < 0.01. **(B)** F (5, 24) = 10.36, *p* < 0.001, **(C)** F (2, 12) = 7.334, *p* = 0.008, **(D)** F (5, 24) = 4.759, *p* = 0.004, **(E)** F (2, 12) = 6.540, *p* = 0.01, one-way ANOVA one-way ANOVA)., **(F)** F (5, 33) = 3.979, *p* = 0.006, **(G)** F (2, 16) = 3.555, *p* = 0.03, **(H)** F (5, 33) = 1.999, *p* = 0.10, **(I)** F (2, 16) = 3.555, *p* = 0.72, one-way ANOVA.

Musclin, NBI 35965 hydrochloride (3100; TOCRIS, Minneapolis, MN, USA), antisauvagine-30 (2071; TOCRIS), and alpha-melanocyte-stimulating hormone (α-MSH, 4057-v; PEPTIDE INSTITUTE. INC., Osaka, Japan) were dissolved in a saline solution (Otsuka Pharmaceutical Factory, Inc., Tokushima, Japan). N-[(1R)-1-[(4-Chlorophenyl)methyl]-2-[4-cyclohexyl-4-(1H-1,2,4-trazol-1-ylmethyl)-1-piperidinyl]-2-oxoethyl]-1,2,3,4-tetrahydro-3-isoquinolinecarboxamide (THIQ, 3031; TOCRIS) was dissolved in dimethyl sulfoxide (DMSO) and diluted in 10% DMSO in saline solution just before the administration. Each solvent was also administered i.p. or i.c.v. as a vehicle (0.1 ml for i.p. and 1 µl for i.c.v.). Musclin and vehicle were administered 15 min before behavioral tests and tissue sampling (**Figure 1A**). Antagonists, agonists, and vehicles were administered intracerebroventricularly 15 min before musclin or vehicle administration.

### 2 10.Water immersion stress

Water immersion (WI) stress was performed as previously described to induce mild depression in mice (37, 38). The floor of the cage was flooded with water at 25 ± 2 °C, to a depth equivalent to ankle height on the mice. The mice were exposed to water for 7 h from 9:00 a.m. to 16:00 p.m. with ad libitum access to food and water, and then returned to their home cage. The WI stress procedure was repeated for 4 consecutive days. Behavioral tests and tissue sampling were performed on the days after the end of the 4-days stress procedure.

### 2.11 Statistical analysis

Data are expressed as mean ± SEM. Comparisons between two groups were performed using a two-tailed Student’s t-test, whereas one-way analysis of variance (ANOVA) followed by Tukey’s Multiple Comparison Test was used to compare three or more groups (GraphPad Prism 10, GraphPad Software, MA, USA). Differences were considered statistically significant at *p* < 0.05.

## 3 Results

### 3.1 Musclin decreased the immobility time and increased the swim distance in the FS test and decreased the open % in the EPM test

To evaluate the effect of musclin on depressive behaviors, the duration of immobility and the swim distance were measured in mice that received i.p. or i.c.v. administration of musclin (**Figure 1A**). The immobility times of mice that were administered 0.1, 0.3, 1, and 3 nmol/mouse i.p. and 0.15 nmol/mouse i.c.v. musclin were significantly lower than those of mice that were administered the vehicle (**Figures 1B, C**). The swim distances of mice that were administered 0.3 nmol/mouse i.p. and 0.15 nmol/mouse i.c.v. musclin were significantly higher than those of mice that were administered the vehicle (**Figures 1D, E**). The values of the open % for mice administered 0.1 and 0.3 nmol/mouse i.p. and 0.15 nmol/mouse i.c.v. musclin were significantly lower than those of mice that were administered the vehicle (**Figures 1F, G**). The values of the total distances of mice administered musclin i.p. or i.c.v. were not different from those of the mice administered with the vehicle (**Figures 1H, I**).

### 3.2 Musclin increased mRNA expression level of urocortin 2 but decreased that of proopiomelanocortin n the hypothalamus

**Figure 2:**
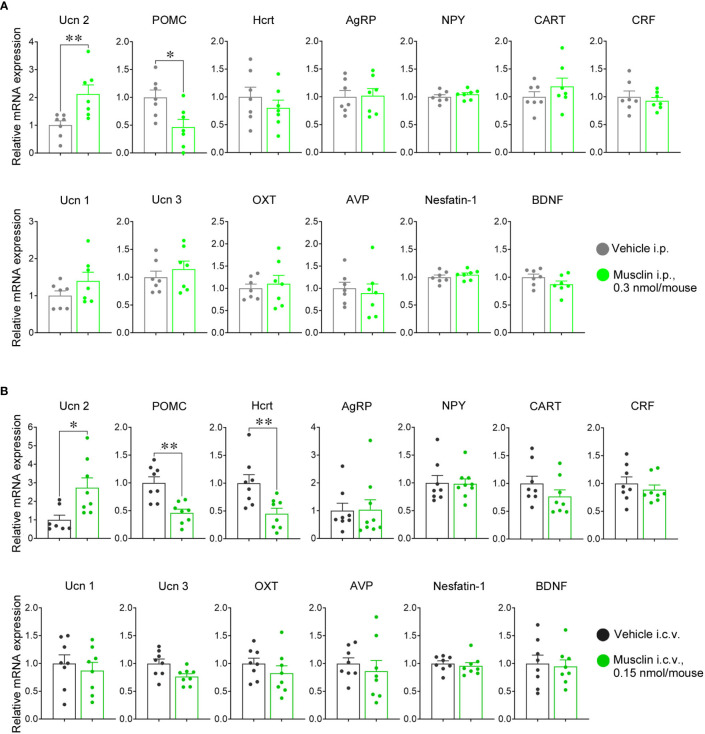
Hypothalamic neuropeptide mRNA levels of mice with intraperitoneal (i.p.) and intracerebroventricular (i.c.v.) administration of musclin. mRNA levels of Ucn 2, POMC, Hcrt, AgRP, NPY, CART, CRF, Ucn 1, Ucn 3, OXT, AVP, Nesfatin-1, and BDNF, were measured in hypothalamus isolated 15 min after i.p. **(A)** and i.c.v. **(B)** administration of musclin or vehicle. Data are represented as means ± SEM (*n* = 7–8). Differences were considered significant at *p < 0.05 and **p < 0.01. **(A)** Ucn 2: t(12) = 3.119, *p* = 0.009; POMC: (t(12) = 2.827, *p* = 0.02, *t*-test; **(B)** Ucn2: t(13) = 2.826, *p* = 0.01; POMC: t(14) = 4.240, *p* < 0.001; Hcrt: t(14) = 3.058, *p* = 0.009, *t*-test. Ucn 2, urocortin 2; POMC, proopiomelanocortin; Hcrt, hipocretin/orexin; AgRP, agouti-related peptide; NPY, neuropeptide Y; CART, cocaine- and amphetamine-regulated transcript; CRF, corticotropin-releasing factor; OXT, oxytocin; AVP, arginine vasopressin; BDNF, brain-derived neurotrophic factor.

To evaluate the alterations in bioactive peptides in the hypothalamus induced by musclin, mRNA expression levels were measured in the PVN of the hypothalamus of mice that were administered 0.3 nmol/mouse i.p. and 0.15 nmol/mouse i.c.v. The treatment changed behaviors in both the FS and EPM tests (**Figures 1B–G**). Tissue sampling was performed according to the schedule (**Figure 1A**). The urocortin 2 (Ucn 2) mRNA levels of mice that were administered musclin i.p. and i.c.v. were significantly higher than those of mice administered the vehicle (**Figures 2A, B**). Proopiomelanocortin (POMC) mRNA expression levels of mice that were administered musclin i.p. and i.c.v. were significantly lower than those of mice administered the vehicle (**Figures 2A, B**). Hypocretin/orexin (Hcrt) mRNA expression levels were lower in mice administered musclin i.c.v. (**Figure 2B**), but there was no alteration with i.p. administration compared to mice administered the vehicle. Other mRNA levels were not altered in mice following i.p. or i.c.v. administration of musclin (**Figures 2A, B**).

### 3.3 I.p. administration of musclin increased c-Fos-positive cells in the PVN and NTS but decreased these cells in the ARC

**Figure 3:**
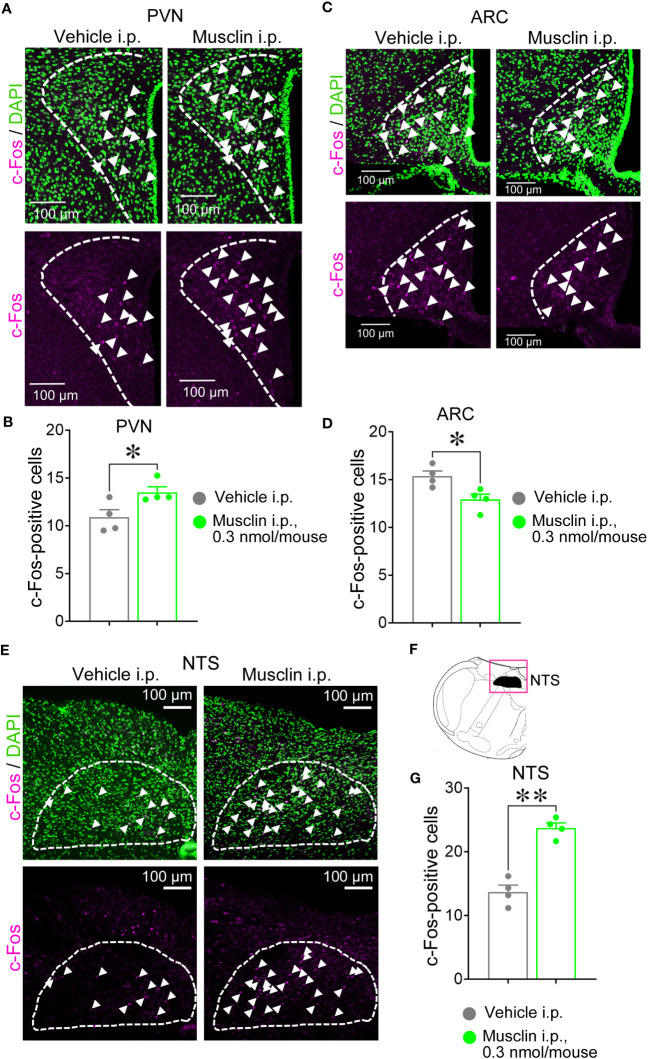
Immunostaining for c-Fos in paraventricular nucleus (PVN) and arcuate nucleus (ARC) in hypothalamus nucleus and tractus solitarii (NTS) of mice with i.p. administration of musclin. Coronal sections were stained with c-Fos antibody. Representative images of c-Fos-positive cells (white arrowheads) in PVN [white dashed line in **(A)**], ARC [white dashed line in **(C)**], and NTS [white dashed line in **(E)**] were obtained using confocal laser microscopy. **(F)** The black color in the magenta square indicates the NTS area in mouse brain atlas. C-Fos-positive cells were counted out on one side of each of six hypothalamic tissues containing PVN **(B)**, ARC **(D)**, and NTS **(G)** per mouse. Data are represented as means ± SEM (*n* = 4). Differences were considered significant at *p < 0.05 and **p < 0.01. PVN: t(6) = 2.632, *p* = 0.04; ARC: t(6) = 3.089, *p* = 0.02; NTS: t(6) = 7.665, *p* < 0.001, *t*-test.

We assessed whether musclin activates the neurons in the PVN and ARC of the hypothalamus, where Ucn 2 and POMC neurons exist, respectively, and the neurons in the NTS, which is a relay point for afferent signals from various peripheral tissues. c-Fos-positive cells were counted in these areas of mice administered with 0.3 nmol/mouse musclin i.p. The number of c-Fos-positive cells was significantly increased in the PVN and NTS but significantly decreased in the ARC compared with those in the same areas of mice administered with the vehicle (**Figures 3A–G**).

### 3.4 c-Fos-positive cells in the PVN induced by i.p. administration of musclin were Ucn 2-positive

**Figure 4:**
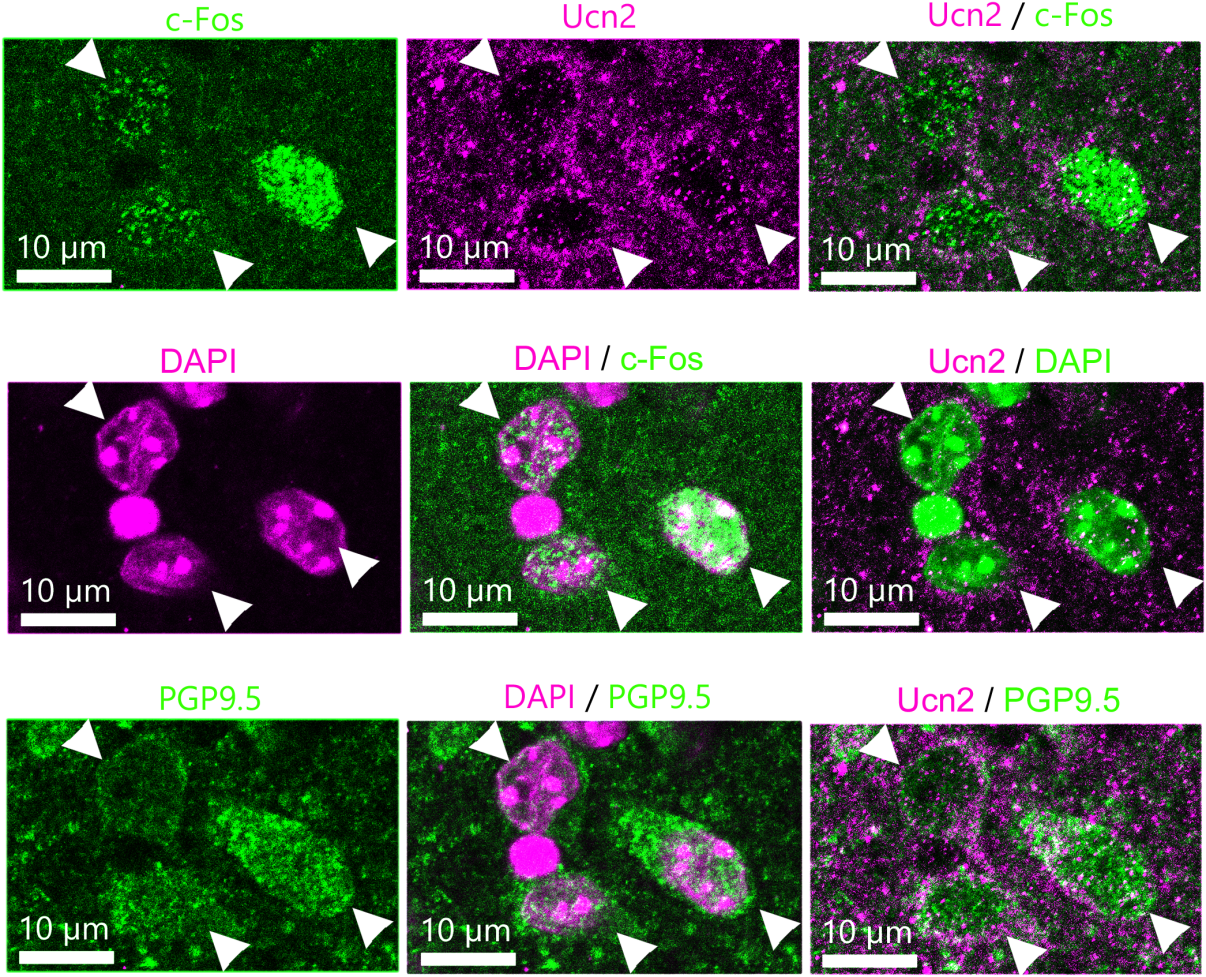
Immunostaining for c-Fos and Ucn 2. Coronal sections of paraventricular nucleus (PVN) were stained with anti-c-Fos antibody, anti-Ucn 2 antibody, anti-PGP9.5 (a pan-neuronal marker) antibody, and DAPI. Representative images of c-Fos-, Ucn2, Ucn 2- and c-Fos-, DAPI-, DAPI and c-Fos, Ucn 2- and DAPI-, PGP9.5, Ucn 2- and PGP9.5, DAPI- and PGP9.5-positive cells in PVN.

To assess whether activated neurons in the PVN of the hypothalamus were Ucn 2-positive, we performed multiple staining with c-Fos, Ucn 2, PGP9.5, and DAPI in the sections of the PVN. In mice that were administered 0.3 nmol musclin i.p., c-Fos-positive cells also showed positivity for Ucn 2 and PGP9.5 (**Figure 4**).

### 3.5 Administration of antisauvagine-30 i.c.v. increased the immobility time and decreased the swim distance, and administration of α-MSH and THIQ i.c.v. increased the open % in mice that were administered musclin

**Figure 5:**
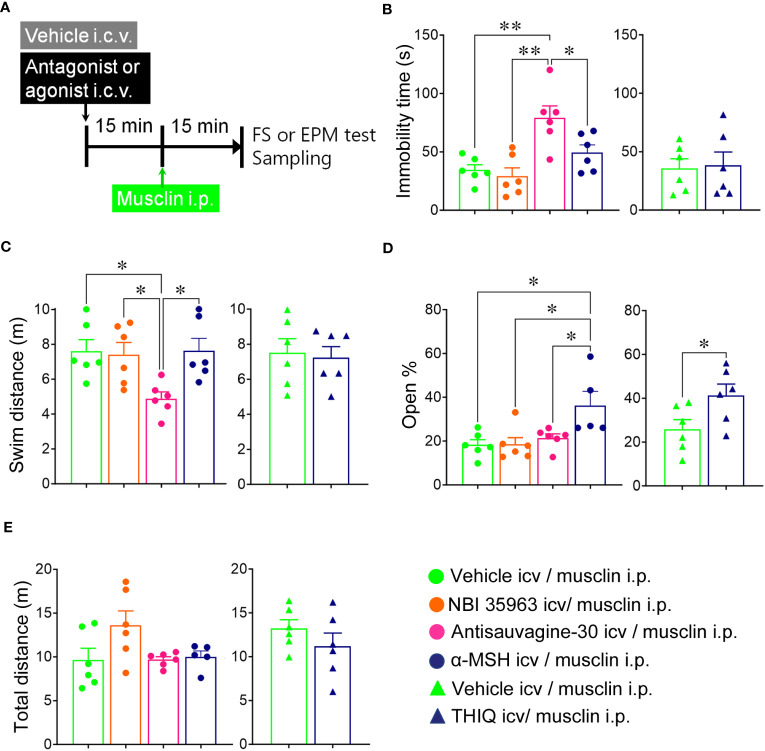
Effects of corticotropin releasing factor (CRF) receptor antagonists and melanocortin receptor (MCR) agonists on changes in behaviors induced by musclin. **(A)** Intracerebroventricular (i.c.v.) administration of selective CRF type 1 receptor antagonist NBI 35963 (5nmol/mouse), selective CRF type 2 receptor antagonist antisauvagine-30 (5 nmol/mouse), MCR agonist α-MSH (24 pmol/mouse), and melanocortin 4 receptor (MC4R) agonist THIQ (5 nmol/mouse), followed by intraperitoneal (i.p.) administration of 0.3 nmol/mouse musclin and the FS test or tissue sampling were performed according to the schedule. The immobility time **(B)** and the swim distance **(C)** in FS test, or open % **(D)** and the total distance **(E)** in EPM were assessed. Data are represented as means ± SEM (*n* = 5-6). Differences were considered significant at *p < 0.05 and **p < 0.01. **(B)** (F (3, 20) = 9.253, *p* < 0.001; **(C)** F (3, 20) = 4.579, *p* = 0.01, **(D)** F (3, 19) = 5.163, *p* = 0.009, one-way ANOVA and t(10) = 2.292, *p* = 0.04,*t*-test.

To assess whether Ucn 2 or POMC contributes to the anti-depressant or anxiogenic effect of musclin, a selective corticotropin-releasing factor (CRF) type 2 receptor antagonist, antisauvagine-30 (5 nmol/mouse, i.c.v.), a selective CRF type 1 receptor antagonist, NBI 35963 (5 nmol/mouse, i.c.v.), a melanocortin receptor (MCR) agonist, α-MSH (24 pmol/mouse, i.c.v.), and a selective melanocortin 4 receptor (MC4R) agonist, THIQ (5 nmol/mouse, i.c.v.) were administered i.c.v. before the i.p. administration of musclin (**Figure 5A**). The mice administered antisauvagine-30 i.c.v., followed by musclin i.p., showed significantly increased immobility times and decreased swim distances (**Figures 5B, C**). The mice administered α-MSH or THIQ i.c.v., followed by musclin i.p., showed significantly increased open % (**Figure 5D**). There were no differences in the total distance among all mice (**Figure 5E**).

### 3.6 Musclin reversed the increase in the duration of immobility and the decreases in the swim distance and the mRNA level of Ucn 2 induced by repeated WI stress in mice

**Figure 6:**
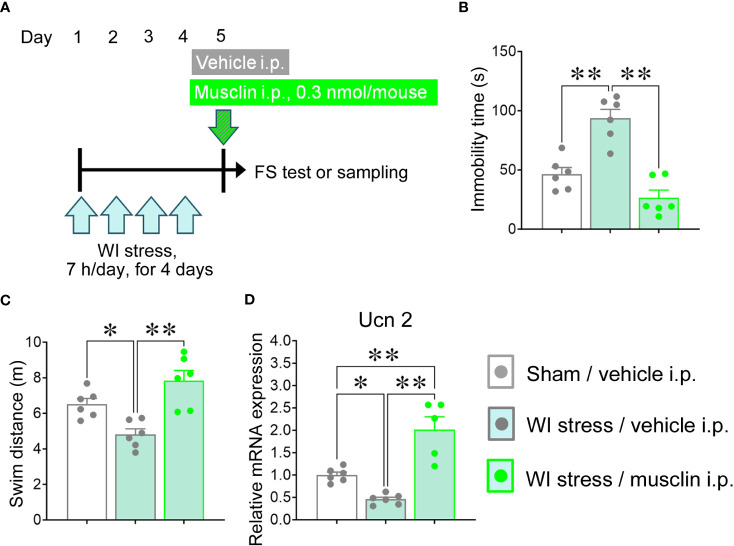
Effect of musclin on depression-like behaviors and the decrease of the mRNA level of Ucn 2 induced by a repeated water immersion (WI) stress. **(A)** Mice were exposed to a repeated WI stress, and forced swim (FS) test and tissue sampling were performed according the schedule. **(B)** The immobility time and the swim distance of repeated WI-stressed mice with intraperitoneal (i.p.) administration of 0.3 nmol/mouse musclin were measured in FS test (*n* = 6). The mRNA level of Ucn 2 was measured in hypothalamus isolated 15 min after administration of musclin or vehicle to different mice than those undergoing the FS tests (*n* = 5–6). Data are represented as means ± SEM. Differences were considered significant at *p < 0.05 and **p < 0.01. **(B)** F (2, 15) = 27.50, *p* < 0.001; **(C)** F (2, 15) = 12.64. *p* < 0.001; **(D)** F (2, 14) = 26.08, *p* < 0.001, one-Way ANOVA.

To assess the anti-depressive effect of musclin in a stress model that induces depression, we applied repeated WI stress, and musclin was administered according to the schedule (**Figure 6A**). The immobility time was significantly increased, and the swim distance was significantly decreased in the FS test upon repeated WI stress in mice, while i.p. administration of musclin reversed these alterations to normal levels observed in sham mice (**Figures 6B, C**). The mRNA level of Ucn 2 in mice exposed to repeated WI stress was significantly low as compared with that in sham mice; however, the level in WI-stressed mice administered with musclin was higher than those in sham mice and WI-stressed mice administered the vehicle (**Figure 6D**). As repeated WI stress did not change the open %, we did not assess anxiety-like behavior in repeated WI-stressed mice in the present study (**Supplementary Figure 1**).

## 4 Discussion

Resistance exercise induces release of musclins (39). Previous studies have reported that in ischemia-reperfusion injury mouse model, musclin knockout cancelled the exercise-induced cardioprotective effects (40, 41). Furthermore, musclin is released by treadmill exercise, enhances exercise endurance, and activates mitochondrial biogenesis (21). Ataman et al. demonstrated that central osteocrin/musclin increased dendritic branch number and complexity in primary human fetal brain culture (42). However, the effects of musclin on emotional behaviors are little known. The depression-like behavior in the FS test was suppressed by both i.p. and i.c.v. administration of musclin in the present study, and an increase in mRNA expression of Ucn 2 was observed in mice administered musclin. Moreover, the anti-depressant effect of musclin was reversed by the CRF type 2 receptor antagonist. The neurons in the hypothalamus, such as AgRP and POMC, respond rapidly to hunger and full situations and alter feeding behavior (43). Neurons in the hypothalamus become activated, increasing mRNA expression, by a 30-minute acute stress (44). Furthermore, central administration of Ucn 2 30 minutes before the FS test session decreased the immobility time of normal mice (45). Hence, hypothalamic neurons can respond quickly against the alterations in the environment. The present study demonstrated that musclin exerted the antidepressant and Ucn 2-activating effects in repeated WI stress. Ucn 2 is a CRF family peptide predominantly expressed in the PVN and supraoptic nuclei of the hypothalamus and locus coeruleus (46). Actions of Ucn 2 such as anorexigenic effect and the increase of motor activity are exhibited through the binding to CRF type 2 receptors, which are distributed in various area of the brain—lateral septal nucleus, bed nucleus, medial nucleus of the amygdala, ventromedial nucleus of the hypothalamus, dorsal raphe, and subcortical structures (46–48). A previous study demonstrated that the central administration of Ucn 2 in mice had an antidepressant effect as demonstrated in the FS test (49). Furthermore, Ucn 2 has been shown to ameliorate depression-like behavior induced in nicotine-withdrawal model mice via the CRF type 2 receptor (50). Our results suggested that musclin might have an anti-depressant effect via the activation of Ucn 2 signaling in the hypothalamus. Therefore, musclin, a new myokine, could be developed as an antidepressant through the activation of Ucn 2 in the hypothalamus, and Ucn 2 might be a new mediator of depression-associated peptides.

Myokines manage the crosstalk between skeletal muscles and the brain (51). Although many factors secreted by peripheral tissues cannot pass through the blood-brain barrier (BBB), some myokines, such as irisin, cathepsin B, BDNF, and IL-6, can pass through the BBB and directly affect cells in the brain (52). The present study demonstrated that both peripheral and central administration of musclin suppressed depression-like behavior, suggesting that musclin functions directly in the brain. However, the number of c-Fos-positive cells in the NTS was increased by the peripheral administration of musclin. The NTS is a relay point for afferent signals from various peripheral tissues, including skeletal muscles, to the brain (53, 54). The present study suggested that musclin can exert an anti-depressant effect through both direct and indirect pathways. This needs to be clarified in future experiments.

Although exogenous administration of Ucn 2 has been reported to have anxiolytic effects in the EPM test, the range of anxiolytic effects of Ucn2 is very narrow (49). Ucn 2-deficient mice have been reported to show no alteration in open % in the EPM test (55). In the present study, musclin induced an increase in the mRNA level of Ucn 2 and exerted an antidepressive effect, and a selective CRF type 2 receptor antagonist canceled its antidepressive effect. However, a selective CRF type 2 receptor antagonist did not alter anxiety-like behaviors induced by musclin. The level of Ucn 2 activated by musclin may mainly contribute to the antidepressant effect, however, it is not believed to induce anxiety.

CRF receptors have been reported to be associated with psychiatric diseases. CRF type 1 receptors in the dorsal raphe nucleus contribute to decreased serotonin release, leading to aggression, while CRF type 2 receptors contribute to stimulating serotonin release, leading to an anti-depressant effect in the nucleus accumbens, an area of stress-related psychiatric disorders (56, 57). The antidepressant effect of musclin might involve the Ucn 2-induced activation of CRF type 2 receptor following to increase in the release of serotonin. Previous studies demonstrated that immobilization stress for 2h, water deprivation stress for 3 days, or maternal separation stress for 13 days (from postnatal day 2 to 14) in rats were enhanced mRNA level of Ucn 2 (58, 59). The differences of Ucn 2 level between these stresses and WI stress might be caused by the differences of stress procedures such as stress stimuli and duration.

In contrast with the increase in Ucn 2 levels, musclin decreased POMC levels and induced anxiety-like behaviors compared to that in vehicle-treated mice. Furthermore, anxiogenic effect of musclin was blocked by central administration of an MC4R agonist. α-MSH is encoded by the POMC gene and is one of the peptides processed from POMC (60). The activation of POMC neurons induced by leptin signaling from adipocytes produces and releases α-MSH and cancels the feeding behavior via MC4R receptors (61). Both the mRNA expression of POMC and the protein concentration of α-MSH in the hypothalamus of obese Zucker rats were lower than those in lean control littermates (62). Thus, the activation of POMC neurons and the secretion of α-MSH are linked. Although the functions of POMC neurons are mainly to control appetite and energy metabolism, an association between POMC neurons and anxiety has also been reported. Cragnolini et al. showed that α-MSH reversed the decrease of open % induced by IL-1 in rats (63). Corda et al. demonstrated that α-MSH reduces punished licking periods in the conflict test in rats (64). Chaki et al. also showed that α-MSH and the MC4R agonist “MT II” reduced punished licking periods in the conflict test in rats (65). Furthermore, the MC4R antagonist “MCL0020” inhibited the decrease in time spent in the light area in a light-dark box test induced by acute swim stress in rats (65). These studies indicate the anxiolytic effect of α-MSH. In contrast, Liu et al. demonstrated that acute stressors, such as restraint stress for 30 min or swim stress for 10 min, increased c-Fos-positive POMC neurons in the ARC, and the melanocortin receptor antagonist “SHU9119” prevented the decrease in entries into the open arm in the EPM test induced by the same acute stressors in rats (66). Gonzalez et al. showed that the infusion of α-MSH into the medial preoptic area induced anxiety-like behavior in the EPM test in female rats (67). Kokare et al. demonstrated that the MC4 receptor antagonist “HS014” suppressed the decrease in the open % in the EPM test induced by ethanol withdrawal in rats (68). These studies indicate the anxiogenic effect of α-MSH. Therefore, it is controversial whether POMC neurons contribute to anxiogenic or anxiolytic effects. In the present study, the decrease in the open % induced by musclin was reversed by the central administration of α-MSH and the MC4R agonist “THIQ,” suggesting that α-MSH has an anxiolytic effect in the present condition.

Hypocretin/orexin has various effects, such as orexigenic effects, sleep regulation, locomotor activation, and opioid abuse, and associations between hypocretin/orexin and psychiatric disorders have been reported (69, 70). It is unclear whether peripheral musclin secreted from the skeletal muscles or administered peripherally as a medication can pass through the BBB. The present study demonstrated that only central administration of musclin decreased the hypocretin/orexin levels, indicating the possibility of a direct effect on the hypocretin/orexin neurons. Ataman et al. have reported that musclin is produced in the human brain but not mice (42). Although it is not clear whether the antidepressant effect of musclin is a direct and/or indirect effect, peripherally administered musclin, as a medication or a supplement, or musclin secreted peripherally by physical activity can at least activate the Ucn 2 signaling. Additionally, the anti-depressant effect of musclin was canceled by antagonizing the CRF type 2 receptor in the present study. The activation of Unc 2 signaling might be predominant in exerting the anti-depressant effects of musclin.

Tanaka et al. proposed that the decrease in swim time in mice exposed to WI stress for 24 h a day for 5 consecutive days reflected fatigue (44). In addition, Yamada et al. demonstrated that mice exposed to WI stress for 14 h a day for three consecutive days had impaired nesting behavior and proposed that this WI stress model is the apathy model (71). A recent study demonstrated that CRF type 2 receptors are present in skeletal muscles and Ucn 2 improves skeletal muscle quality and function in normal and obese mice (72, 73). In the present study, administration of musclin to intact mice increased the swim distance, activation of Ucn 2; however, more musclin reversed the decrease in swim distance and Ucn 2 level induced by WI stress. These results partially reflect the anti-fatigue effects of musclin (21).

The present study has certain limitations. We did not use target gene silencing or knockout mice. We did not identify the projection target of Ucn 2 neurons in the hypothalamus. Further, it has been reported that there are gender differences in the risk of developing depression (74). We need to further explore the function of musclin in mental diseases involving sex-differences and the muscle-brain axis. The present results should be validated in future clinical studies. However, the present study is the first to demonstrate that musclin exerts an antidepressant effect by upregulation Ucn 2 in the hypothalamus. Musclin and Ucn 2 signaling may be novel targets for the treatment of depression and for elucidating the mechanism of the muscle-brain axis.

## Data availability statement

The original contributions presented in the study are included in the article/**Supplementary Material**. Further inquiries can be directed to the corresponding author.

## Ethics statement

The animal study was approved by Kobe Pharmaceutical University Committee for Animal Experiments (approval no. 2023-011 and -014). The study was conducted in accordance with the local legislation and institutional requirements.

## Author contributions

KA: Data curation, Formal Analysis, Investigation, Methodology, Project administration, Writing – original draft. AA: Conceptualization, Funding acquisition, Writing – review & editing. HI: Investigation, Writing – review & editing. IK: Conceptualization, Investigation, Writing – review & editing.

## Supplementary material

Supplementary Figure 1 The values of open % and total distance in repeated water immersion (WI)-stressed mice with i.p. administration of vehicle. Time spent in open arm and total distance of mice 24 h after WI stress for 4 days. The relative open time ratio (open %) was calculated as described in the Methods. Data are represented as means ą SEM (n = 8).
