# Supplementary figures and images for: Musclin prevents depression-like behavior in male mice by activating urocortin 2 signaling in the hypothalamus
Source: Front Endocrinol (Lausanne). 2023 Dec 5;14:1288282. doi: 10.3389/fendo.2023.1288282 (PMC10728487; doi:10.3389/fendo.2023.1288282)

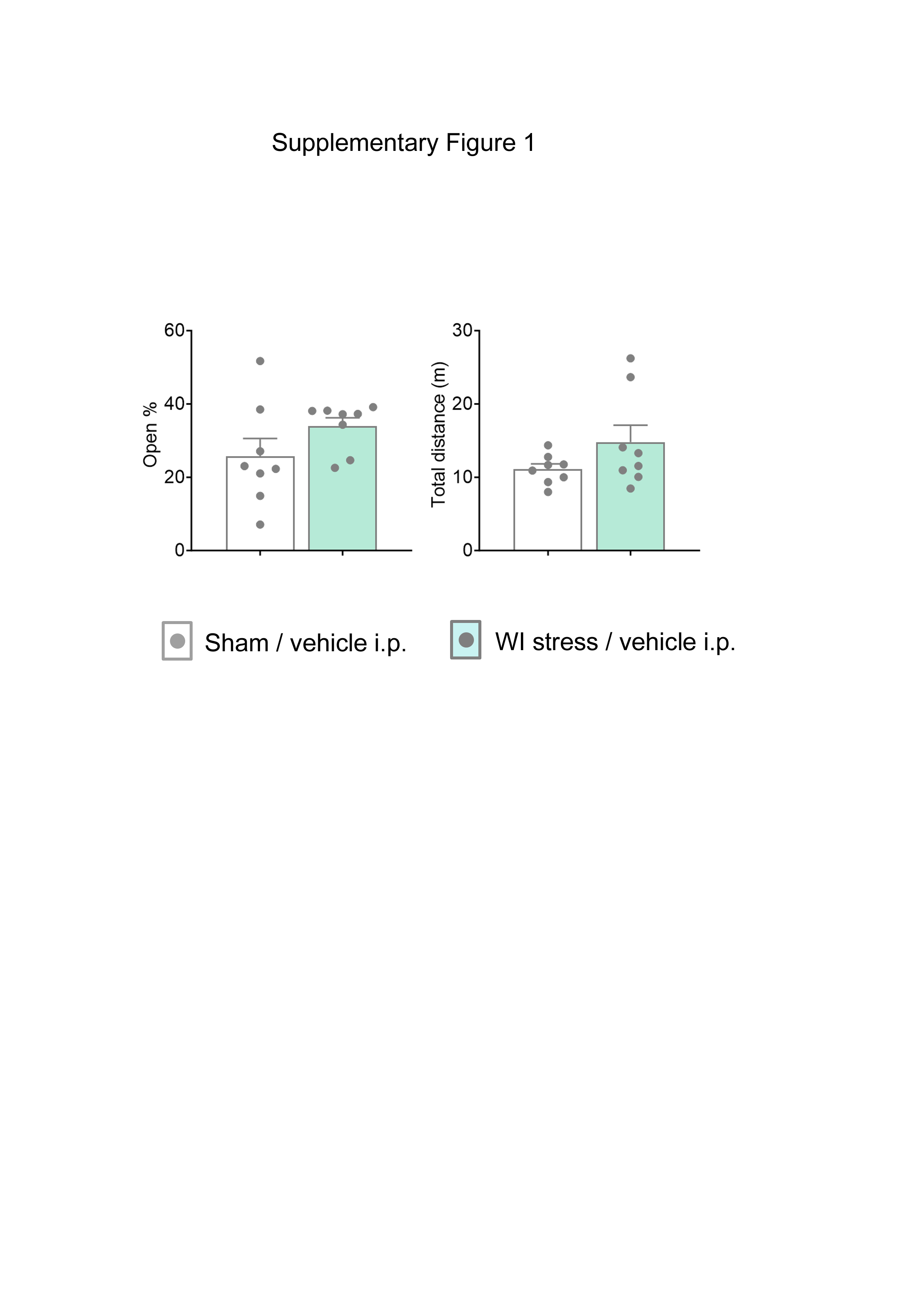

Supplement: Supplementary Figure 1 — The values of open % and total distance in repeated water immersion (WI)-stressed mice with i.p. administration of vehicle. Time spent in open arm and total distance of mice 24 h after WI stress for 4 days. The relative open time ratio (open %) was calculated as described in the Methods. Data are represented as means ą SEM (n = 8). [file Image_1.tif]
