# Supplementary material for: Musclin prevents depression-like behavior in male mice by activating urocortin 2 signaling in the hypothalamus
Source: Front Endocrinol (Lausanne). 2023 Dec 5;14:1288282. doi: 10.3389/fendo.2023.1288282 (PMC10728487; doi:10.3389/fendo.2023.1288282)
Supplement: Supplementary file 2 [file Table_1.docx]

Supplementary table 1. The primers used for real-time qPCR (ID number)

Forward Reverse

urocortin 2 (Ucn 2, MA206860, Takara) NM_145077 TTGGCCAAACACATACCCAGA GCTCAGAAGCATGGCAAGACAC

proopiomelanocortin (POMC, MA227485, Takara) NM_001278581 TGCGCATCTTAGCAGATCTGG TGCAGAGGCAAACAAGATTGG

hypocretin/orexin (Hcrt, Bio Rad) NM_010410 no information provided

agouti-related peptide (AgRP, MA158464, Takara) NM_001271806 GGACTGAGCATAAAGATGGCATGA TGTAGCCAGGGCATGAGGTG

neuropeptide Y (NPY, MA233422, Takara) NM_023456 GCTCTGCGACACTACATCAATCT GATGAGGGTGGAAACTTGGAA

cocaine- and amphetamine-regulated transcript NM_013732 GACATCTACTCTGCCGTGGATGA TTCTTGCAACGCTTCGATCTG

(CART, MA100744, Takara)

corticotropin-releasing factor (CRF, CM001098, Takara) NM_20576 AACGATTCTGCATTTAGCACACAAG TGATATCGGAGCTGCGATATGG

urocortin 1 (Ucn 1, Bio Rad) NM_021290 no information provided

urocortin 3 (Ucn 3, MA22325, Takara) NM_031250 AGCATTTCCACTCCAGAGCAAAG AGCTGAAGACTGGTCCAGTGTTGTA

oxytocin (OXT, Bio Rad) NM_011025 no information provided

arginine vasopressin (AVP, Bio Rad) NM_00973 no information provided

nesfatin-1 (MA206151, Takara) NM_001360375 GGCGCAGAAGCAGGAGTATCA TTCCAAGTTCTGGCAGACATCA

brain-derived neurotrophic factor NM_001048139 AGTTGGAAGCCTGAATGAATGGA CTGATGCTCAGGAACCCAGGA

(BDNF, MA23602, Takara)

glyceraldehyde-3-phosphate dehydrogenase NM_008084 TGTGTCCGTCGTGGATCTGA TTGCTGTTGAAGTCGCAGGAG

(GAPDH, MA050371, Takara)
